# Circadian rhythm and its role in malignancy

**DOI:** 10.1186/1740-3391-8-3

**Published:** 2010-03-31

**Authors:** Sobia Rana, Saqib Mahmood

**Affiliations:** 1 Department of Physiology & Cell Biology, University of Health Sciences, Lahore, Pakistan; 2 Department of Human Genetics & Molecular Biology, University of Health Sciences, Lahore, Pakistan

## Abstract

Circadian rhythms are daily oscillations of multiple biological processes directed by endogenous clocks. The circadian timing system comprises peripheral oscillators located in most tissues of the body and a central pacemaker located in the suprachiasmatic nucleus (SCN) of the hypothalamus. Circadian genes and the proteins produced by these genes constitute the molecular components of the circadian oscillator which form positive/negative feedback loops and generate circadian rhythms. The circadian regulation extends beyond clock genes to involve various clock-controlled genes (CCGs) including various cell cycle genes. Aberrant expression of circadian clock genes could have important consequences on the transactivation of downstream targets that control the cell cycle and on the ability of cells to undergo apoptosis. This may lead to genomic instability and accelerated cellular proliferation potentially promoting carcinogenesis. Different lines of evidence in mice and humans suggest that cancer may be a circadian-related disorder. The genetic or functional disruption of the molecular circadian clock has been found in various cancers including breast, ovarian, endometrial, prostate and hematological cancers. The acquisition of current data in circadian clock mechanism may help chronotherapy, which takes into consideration the biological time to improve treatments by devising new therapeutic approaches for treating circadian-related disorders, especially cancer.

## Introduction

In humans, like other organisms, most physiological and behavioral functions are manifested rhythmically across days and nights. All healthy human beings exhibit the common attribute of sleeping at night and waking up in the morning automatically. When a human being encounters a new day, the body prepares itself for the new tasks ahead and boost heart rate, blood pressure and temperature. On the other hand, the same parameters decline at the end of the day. Such daily occurring rhythms with a period of about 24 hours are termed as circadian (from the Latin "*circa diem" *meaning "about a day") rhythms [1]. These rhythms are the outward manifestation of an internal timing system generated by a circadian clock that is synchronized by the day-night cycle [2].

## Circadian clocks

The circadian timing system proficiently coordinates the physiology of living organisms to match environmental or imposed 24-hour cycles [3]. Circadian clocks are endogenous and self-sustained (meaning that rhythms can continue even in the absence of external cues) time-tracking systems that enable organisms to anticipate environmental changes, thereby adapting their behavior and physiology to the appropriate time of day [4]. This provides organisms with an anticipatory adaptive mechanism to the daily predictable changes in their environment such as light, temperature and social communication, and serves to synchronize multiple molecular, biochemical, physiological and behavioral processes. A wide range of biological processes are regulated by the circadian clock including sleep-wake cycles, body temperature, energy metabolism, cell cycle and hormone secretion [5,6].

## Central pacemaker or the master clock

The mammalian clock system is hierarchical with a master clock that controls circadian rhythms and resides in the suprachiasmatic nucleus (SCN) of the hypothalamus. Damage to the SCN can render experimental animals arrhythmic and cause sleep disorders in patients. Moreover, intracerebral grafts of perinatal SCN can reinstate behavioural circadian rhythms of SCN-ablated rodents [7]. The SCN pacemaker consists of multiple, autonomous single cell circadian oscillators, which are synchronized to generate a coordinated rhythmic output in intact animals [8,9]. In mammals, the circadian photoreception pathways are distinct from those of visual perception [10-12]. Light is perceived by a subset of melanopsin-expressing retinal ganglion cells, and the photic information is directly conveyed to the SCN clock through the retino-hypothalamic tract [13-15]. This photic entrainment corrects the phase of the SCN oscillator every day to ensure synchronization of circadian with geophysical time. The phase of SCN rhythms can be shifted by exposure of the animal to a new light/dark schedule or to short light pulses during the subjective night [16]. Entrainment of a biological clock is the process of determining both its period (which is 24 hours in most humans) and its phase. The latter refers to the offset of a circadian clock with respect to the standard 24-hour cycle. In general terms, the period of the clock is genetically determined, whereas its phase is heavily influenced by environmental zeitgebers (cues or stimuli) such as light.

## Peripheral oscillators or the slave oscillators

A major finding in the field of circadian rhythms in recent years is that the SCN is not the only circadian clock in the organism. Indeed, most tissues including extra-SCN brain regions and peripheral organs bear circadian oscillators [17]. Moreover, these extra-SCN oscillators can function independently from the SCN [18]. Peripheral mammalian cell types contain functional circadian oscillators, but these may not respond to light-dark cycles and can be entrained by non-photic stimuli [19,20]. These circadian oscillators are sensitive to a variety of chemical cues or to temperature cycles [21,22]. The SCN synchronizes peripheral clocks in organs such as liver, heart, and kidney via indirect and direct routes so that a coherent rhythm is orchestrated at the organismal level to ensure temporally coordinated physiology [23-25]. Indirect synchronization is achieved by controlling daily activity-rest cycles and, as a consequence, feeding time. Feeding (or starving) cycles are dominant zeitgebers for many, if not most, peripheral clocks. Food metabolites, such as glucose, and hormones related to feeding and starvation are probably the feeding-dependent entrainment cues. Activity cycles also influence body temperature rhythms, which in turn can participate in the phase entrainment of peripheral clocks. Direct entrainment may employ cyclically secreted hormones and perhaps neuronal signals conveyed to peripheral clocks via the peripheral nervous system. Body temperature rhythms, which are controlled in part by the SCN, may also contribute to the synchronization of peripheral clocks [26].

## Molecular mechanism of the circadian clock

**Figure 1:**
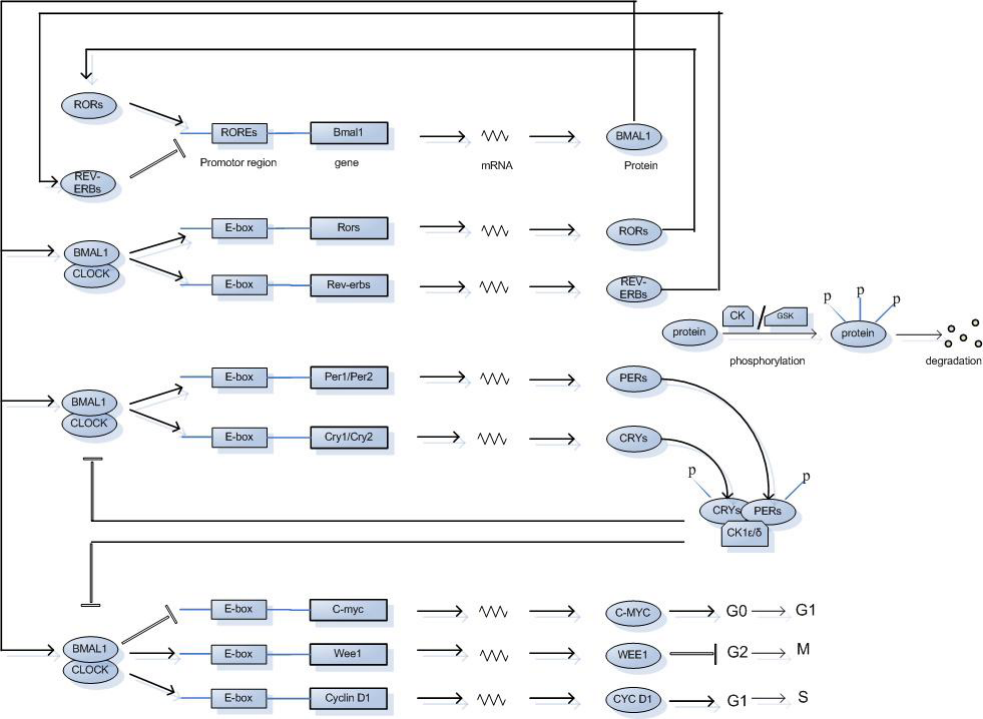
**Schematic representation of the mammalian circadian clock mechanism**. ROREs are retinoic acid-related orphan nuclear receptor response elements present in *Bmal1 *promoter to which REV-ERBs and RORs compete to bind whereas E-boxes are regulatory enhancer sequences present in the promoter regions of the genes under consideration to which CLOCK-BMAL1 heterodimer binds. Casein kinase (CK) isoforms phosphorylate PER, CRY and BMAL1 proteins decreasing their stability and critically regulating the time of action of clock proteins. Similarly, targets of GSK3 (glycogen synthase kinase-3) include PER, REV-ERBα and CRY2. *c-Myc, Wee1 *and *Cyclin D1 *are clock-controlled cell cycle genes.

The clock mechanism in the SCN and the peripheral oscillators is known to be similar at the molecular level [27]; however, the output pathways elicited can be different and more tissue specific. The molecular clockwork is composed of a network of transcriptional-translational feedback loops (Fig. 1) that drive rhythmic, ~24-hour expression patterns of core clock components [28]. Core clock components are genes whose protein products are necessary for the generation and regulation of circadian rhythms within individual cells throughout the organism [29]. The core clock components include two gene families: *Period *and *Cryptochrome*. In mammals, the expression of three *Period *genes (*Per1, Per2 *and *Per3*) and two *Cryptochrome *genes (*Cry1 *and *Cry*2) is activated by a dimer of the proteins CLOCK (Circadian Locomotor Output Cycles Kaput) and BMAL1 (Brain-Muscle Arnt-Like protein 1). CLOCK and BMAL1 are transcriptional factors that heterodimerize and induce the expression of *Per *and *Cry *genes by binding to their promoters at E-boxes [28,30,31]. CLOCK also has an intrinsic histone acetyltransferase (HAT) activity, thereby it can induce chromatin remodeling and create a permissive state for activation of gene expression [32]. PER and CRY proteins are synthesized in the cytoplasm and they associate before entering the nucleus. In the nucleus, CRYs repress the activity of CLOCK and BMAL1 and in this way, they negatively feedback on their own expression [33,34]. However, the exact molecular mechanism of this repression is yet unclear. The enzymatic activity of CLOCK also allows it to acetylate non-histone substrates. For example, CLOCK mediates acetylation of its own binding partner, BMAL1, on Lys537. Ectopic expression of wild-type BMAL1, but not an acetylation-resistant BMAL1 mutant (K537R), is able to rescue the circadian expression of endogenous target genes in mouse embryonic fibroblasts (MEFs) derived from *Bmal1*^-/- ^mice. The BMAL1-K537R mutant has drastically reduced sensitivity to CRY1-mediated repression compared with wild type BMAL1, indicating that the acetylation of BMAL1 by CLOCK might be an essential regulatory switch as it facilitates CRY-dependent repression [35]. Another crucial modulator of the circadian clock machinery identified recently is a histone deacetylase, namely sirtuin 1 (SIRT1), which regulates circadian rhythms by counteracting the HAT activity of CLOCK [36]. SIRT1 is required for high-magnitude circadian transcription of several core clock genes, including *Bmal1, Rorγ, Per2, and Cry1*. SIRT1 binds CLOCK-BMAL1 in a circadian manner and promotes the deacetylation and degradation of PER2 [37].

CLOCK-BMAL1 heterodimers induce a second regulatory loop activating transcription of retinoic acid-related orphan nuclear receptors, *Rev-erbα *and *Rorα *[38]. Both of these proteins are transcription factors that bind to the *Bmal1 *promoter at REV-ERBα and RORα response elements. RORα activate transcription of *Bmal1 *[39,40], whereas REV-ERBα repress the transcription process [41,42]. Another core member of the mammalian circadian clock is neuronal PAS-domain protein 2 (NPAS2). NPAS2 is a paralogue of CLOCK, exhibiting similar activities but differing in tissue distribution. NPAS2 can heterodimerize with BMAL1, bind to E-box motifs and transcriptionally activate circadian genes [43].

The feedback loops described above are responsible for varying levels of messenger ribonucleic acids (mRNAs) from the *Per, Cry, Rev-erbα *and *Bmal1 *genes across circadian phases. In the SCN, *Per, Cry *and *Rev-erbα *all exhibit a peak of abundance during the light phase, while *Bmal1 *has an opposite phase (i.e., peaks about 12 hour later). In most other brain regions and peripheral tissues, these rhythms are all delayed by several hours but generally keep a similar phase relationship amongst them. In some brain regions, PER oscillations are in phase with those seen in the SCN [44,45]. Considering that simple transcriptional feedback loops like those described above would normally lead to mRNA oscillations with a period much smaller than 24 hours, other mechanisms have been added onto this simple loop model to permit a slowing down and delay of its progression that create a coordinated molecular cycle approximating the 24 hours environmental period. These mechanisms act at different levels involving post-transcriptional processing of the mRNAs, translation, post-translational processing of the proteins and nuclear translocation [46-48]. Each of these can individually contribute to introduce the delay between the activation and repression of transcription that is required to keep the period at ~24 hours.

The post-translational modifications regulating the circadian clock include acetylation, phosphorylation, ubiquitination and sumoylation. In terms of phosphorylation, Casein kinase 1 epsilon (CK1ε) and Casein kinase 1 delta (CK1ε and CK1δ), Casein Kinase 2 (CKII), glycogen synthase kinase-3 (GSK3) and adenosine monophosphate-activated protein kinase (AMPK) are critical factors that regulate the core circadian protein turnover. It has been shown that mutations in *CK1ε *and *CK1δ *result in altered kinase activities and cause shorter circadian periods in mammals [49]. BMAL1 and CRYs are reported to be the targets of CKIε [50]. Casein Kinase 2 (CKII) is one of the more recent kinases identified as a clock component in *N. crassa *[51,52] and *D. melanogaster*. [53-55] but its role in regulation of mammalian clock has yet to be clarified. Changes in GSK3 activity have been reported to alter period length in mammalian cells [56]. The targets of GSK3 in mammals might be the PER proteins (PER phosphorylation by GSK3 might prevent nuclear entry of PER proteins), REV-ERBα [57] and/or CRY2 (for which phosphorylation might control CRY2 degradation at the end of night) [58]. Recently, the nutrient-responsive AMPK has been found to regulate circadian clock by phosphorylation and destabilization of the clock component CRY1 [59]. Also, AMPKγ3 subunit is found to be involved in the regulation of peripheral circadian clock function [60]. Likewise kinases, phosphatases also participate in clock regulation. Most recently, the serine/threonine phosphatase PP5 (protein phosphatase 5) has been found to interact with and be regulated by CRY proteins [61]. Through its interaction with CRY, PP5 might regulate the phosphorylation state and so the activity of CKIε in the clock [62,63]. Thus, it can be said that phosphorylation by kinases, balanced by regulated dephosphorylation, sets the stage for protein degradation.

Phosphorylation is required for the recruitment of ubiquitin ligases, which mediate the polyubiquitylation and the subsequent degradation of these proteins in the proteasome. In mammals, the stability of PER1 and PER2 is regulated by either βTrCP1 or βTrCP2. CKI phosphorylates PER1 and PER2 and this phosphorylation leads to the recruitment of βTrCP which mediates the ubiquitylation and proteasomal degradation of these proteins [64,65]. Most recently, sumoylation has been revealed as an additional level of regulation within the core mechanism of the circadian clock. It is a reversible posttranslational modification in which a small ubiquitin-related modifier protein (SUMO) is covalently linked to lysine residues. It is controlled by an enzymatic pathway analogous to the ubiquitin pathway. BMAL1 has been found to be rhythmically sumoylated *in vivo *through a process that requires the heterodimerization partner CLOCK. Sumoylation of BMAL1 regulates the turnover of the protein, as a mutation in the sumoylation site (K259R) of BMAL1 lengthens the half-life of BMAL1 [66]. However, SUMO ligases and proteases which may be involved in controlling this sumoylation and their circadian regulation are still to be known.

The transcriptional circadian regulation extends beyond core clock components to include various clock-controlled genes (CCGs), i.e., genes that are under the direct or indirect transcriptional control of the clock transcription factors but are not themselves part of the clock. Regulation of clock-controlled genes is a mechanism by which the molecular clockwork controls physiological processes. The clock-controlled genes (CCG) constitute about 10% of the expressed genes in a given tissue (SCN or in peripheral tissues) to generate rhythmic outputs, and, apart from few exceptions, most of these clock-controlled genes are distinct in different tissues depending upon different physiological needs [67]. Clock-controlled genes may encode a variety of proteins including key regulators for cell cycle.

## Circadian clock and cell cycle

Circadian clock and cell cycle are global regulatory systems found in almost all organisms. The circadian clock shares a number of conceptual and molecular similarities with the cell cycle [68]. Both are periodic for ca. 24 hours, and intrinsic to most cells. Similarly, both are based on the conceptual device of interlock auto-regulatory loops. Moreover, both rely on sequential phases of transcription, translation and protein modification and degradation. The circadian clock controls the expression of cell cycle-related genes; in contrast, circadian clockwork can oscillate accurately and independently of the cell cycle, [69]. It is thereby highly relevant that CCGs include genes that play an essential role in cell cycle control.

It has been shown that CLOCK-BMAL1 directly regulate cell cycle genes such as *Wee1 *(G2-M transition) [69], *c-Myc *(G0-G1 transition) and *Cyclin D1 *(G1-S transition) [70]. The level of antimitotic WEE1 kinase in the liver of *Cry *mutant mice (cryptochromeless mice) is found to be elevated and consequently, liver regeneration in these mice following partial hepatectomy is delayed relative to wild-type controls [69]. The binding of CLOCK-BMAL1 to the E-boxes of *Wee1 *promoter stimulates the transcription of this gene. The elevation of WEE1 in the *Cry *mutant is ascribed to the lack of inhibition of CLOCK-BMAL1 by CRY [69,71]. WEE1 is a cell cycle kinase that plays a key role in the G2-M transition. Ongoing DNA replication or the presence of DNA damage activate WEE1, which then phosphorylates CDC2 (cell division cycle 2)/Cyclin B1 complex, causing its inactivation and delay of mitosis or arrest of the cell cycle at the G2-M interface [72,73]. It is conceivable that elevated WEE1 in *Cry *mutant mice phosphorylates CDC2/CYCB1 complex at an increased rate even in nonstressed cells, slowing down the G2-M transition and the overall growth rate [69].

Transcription of *c-Myc*, which plays an important role in both cell proliferation and apoptosis, is found to be upregulated, and transcription of *p53*, which plays a critical role in the G1-S checkpoint, is downregulated in *Per2 *mutant mice (*mPer2*^*m*/*m*^). Also, there is a general cell cycle dysregulation as the circadian expression pattern of genes functioning in cell proliferation and tumour suppression, such as *Cyclin D1*, *Cyclin A*, *Mdm-2 *(murine double minute, a negative regulator of p53) and *Gadd45α *(growth arrest and DNA damage-inducible protein α), is deregulated. Consequently, these animals have increased incidence of spontaneous and ionizing radiation-induced lymphomas and an increased rate of mortality after ionizing radiation [70]. Normally, the binding of CLOCK-BMAL1 to the E-boxes of *c-Myc *promoter inhibits the transcription of this gene. Upregulation of *c-Myc *transcription in *Per2 *mutant is ascribed to the reduced level of BMAL1 because PER2, in addition to its inhibitory effect on the CLOCK-BMAL1 complex, stimulates transcription of the *Bmal1 *gene [28,74]. Oncogenic transformation mediated by c-*Myc *must overcome its proapoptotic activity [75] in which modulation of p53-mediated apoptosis plays an important role [76,77]. Overexpression of *c-Myc *can induce genomic DNA damage and compromise p53 function, presumably through a reactive oxygen species (ROS)-mediated mechanism [78]. Following γ radiation, MYC-overexpressing cells are less efficient in G1 arrest compared to normal cells [79,80], indicating that c-*Myc *overexpression could drive cells to progress through cell cycle in the presence of genomic DNA damage. Following γ radiation, the loss of *mPer2 *function partially impairs p53-mediated apoptosis, leading to accumulation of damaged cells. However, the mutant *mPer *cells, expressing MYC at elevated levels, could still progress through cell cycle in the presence of genomic DNA damage, resulting in the high incidence of tumor development after γ radiation.

*Cyclin D1 *(*CCND1*) is also a clock-controlled cell cycle gene. Overexpression of *CCND1 *induces mammary tumorigenesis, in addition, increased levels of CCND1 in ERα (estrogen receptor α)-positive breast cancer is associated with poor prognosis [81]. However, additional studies are needed to know whether the rhythmic expression of CCND1 is deregulated in cancer.

Recently, it has been reported that *p21 *(*Waf1/Cip1*), which does not possess an E-box element in its regulatory region, is controlled indirectly via CLOCK/BMAL1-mediated transcriptional regulation of the orphan nuclear receptor *Rev-erb*. *p21 *circadian expression is dramatically increased and no longer rhythmic in *Bmal1 *knock-out mice. *p21 *upregulation in *Bmal1*^-/- ^animals primarily results from the loss of *Rev-erb*α and *Reverb*β expression possibly combined with the increased expression of *ROR*γ [82]. In this context, the release of the REV-ERB-dependent inhibition of RORα4 activity is also likely to play a role. Changes in additional unidentified positive and negative regulators of *p21 *expression may also play an additional role. Thus, in liver, the clock control of *p21 *high amplitude oscillation results from a *RORa4*- and *ROR*γ-dependent activation, which is rhythmically repressed by REV-ERBα and REV-ERBβ. As *p21 *negatively regulates cell cycle progression by inhibiting the activity of CYCE/CDK2 complexes during G1 phase progression, p21 overexpressing *Bmal1*^-^/^- ^primary hepatocytes exhibit a decreased proliferation rate [82].

## Circadian clock, DNA damage response and tumour suppression

The circadian control of an organism's response to DNA damage response rests upon circadian proteins which play important roles in the processes of cell proliferation and control of response to genotoxic stress both at the cellular and organismal levels [83]. DNA damage triggers cellular stress response pathways which may result in checkpoint cell cycle arrest, apoptosis or DNA repair. DNA damage leads to activation of critical components of cellular stress response pathways including ATM/ATR (ataxia telangiectasia mutated/ataxia telangiectasia and Rad3-related) and CHK1/2 (checkpoint kinase1/2) which in turn activates tumour suppressor protein p53 and subsequently causes cell cycle arrest or apoptosis [84]. It has been shown that *Bmal1*-deficient human cells are unable to undergo growth arrest on *p53 *activation by DNA damage. Contrary to *in vivo *mouse data connecting BMAL1-dependent delay in G1 progression to upregulation of *p21 *[82], radiation induced growth arrest in *Bmal1*-deficient human cells correlated with the decrease in levels of p53 and p21 [85]. This disparity may be attributable to interspecies variation or differences between in vitro and in vivo state and warrants further investigation.

PER1 seems to function as a tumour suppressor by regulating cell cycle genes and interacting with key DNA damage-activated checkpoint proteins. *Per1 *overexpression in cancer cells increases ionizing radiation-induced apoptosis, whereas inhibition of *Per1 *in similarly treated cells blunts apoptosis. Ionizing radiation leads to PER1 nuclear translocation, the induction of c-*Myc *expression and repression of *p21 *(*Waf1/Cip1*). Moreover, PER1 directly interacts with the DNA double-strand break-activated kinases ATM and CHK2. Thus, PER1 can function as a tumour suppressor by activating multiple pathways, including the DNA damage response [86]. Another circadian protein, timeless (TIM), which is necessary for the robustness of rhythmicity [87], has been shown to interact with the cell cycle checkpoint proteins ATR, CHK1 and ATRIP (ATR-interacting protein). This interaction is also stimulated by DNA damage, and TIM seems to function as a mediator between sensors and effectors of the DNA damage response [88].

PER2 protein has also been proposed to function as a tumor suppressor. *Per2 *mutant mice develop γ radiation-induced lymphomas at a higher rate than wild-type controls due to partial impairment of p53-mediated apoptosis [70]. Moreover, crossing these mice with polyp formation-prone adenomatosis polyposis coli (*Apc*)^*Min*/+ ^animals increases the frequency of formation of intestinal and colonic polyps in *Apc*^*Min*/+^*Per2*^*m*/*m *^mice compared to *Apc*^*Min*/+ ^mice. Following downregulation of *Per2*, *Cyclin D*, which is a circadian regulated and β-catenin target gene, has been shown to increase in human colon cancer cell lines, as does cell proliferation. Thus, *Per2 *loss during intestinal tumorigenesis may, in part, act through upregulation of β-catenin, increasing intestinal β-catenin signaling and cell proliferation. Also, increase in small-intestinal mucosa β-catenin in *Per2*^*m*/*m *^mice is associated with an increase in MYC protein, again a circadian regulated and β-catenin target gene [89]. Furthermore, accelerated β-catenin expression is associated with PER2 protein instability and lower PER2 levels as a result of increased β-TrCP protein levels as it has been observed that overexpression of wild type or mutant β-catenin protein decreases the stability of PER2 protein, and this PER2 instability is reversed when the induction of β-TrCP is prevented [90]. It has also been reported that *mPer2 *may play an important role in tumor suppression by inducing apoptotic cell death. Overexpression of *Per2 *in the mouse Lewis lung carcinoma cell line (LLC) and mammary carcinoma cell line (EMT6) results in reduced cellular proliferation and rapid apoptosis, but not in non-tumorigenic NIH3T3 cells. This is attributable to enhanced proapoptotis signaling and attenuated anti-apoptosis processes as overexpressed mPER2 downregulate the mRNA and protein levels of *c-Myc*, *Bcl-XL *and *Bcl-2*, and upregulate the expression of *p53 *and *bax *in *mPer2*-overexpressing LLC cells [91]. Similarly, the intratumoral expression of *mPer2 *in C57Bl/6J mice transplanted with Lewis lung carcinoma shows a significant antitumor effect [92]. All this evidence indicates that *Per2 *has a role in tumour suppression, but further research is needed to ascertain whether *Per2 *is in fact a tumour suppressor gene or whether a particular mutation of *Per2 *acquires oncogenetic properties.

In contrast to *Per2 *mutants, *Cry *double mutant (*Cry1*^-/-^*Cry2*^-/-^) mice are indistinguishable from the wild-type controls with respect to radiation-induced morbidity and mortality. Similarly, the *Cry1*^-/-^*Cry2*^-^/^- ^mutant fibroblasts are indistinguishable from the wild-type controls with respect to their sensitivity to ionizing radiation and UV radiation, and ionizing radiation-induced DNA damage checkpoint response [93]. In another study, mice deficient in the core circadian gene *Bmal1 *show reduced lifespan and various symptoms of premature aging but none of the *Bmal1*^-/- ^mice develop tumors in the course of their lifespan [94]. Similarly, *Clock/Clock *mutant mice do not display predisposition to tumor formation either during their normal lifespan or when exposed to a low dose of γ-radiation that is able to initiate and promote neoplastic progression [95]. Instead, exposure of *Clock*^-/- ^mice to ionizing radiation results in the development of pathological conditions similar to those of premature aging described for *Bmal1*^-/- ^mice [94]. Recently, Ozturk *et al*. reported the effect of the *Cry *mutation on carcinogenesis in a mouse strain prone to cancer because of a *p53 *mutation. Contrary to the expectation that clock disruption in this sensitized background would further increase cancer risk, they found that the *Cry *mutation protects *p53 *mutant mice from the early onset of cancer and extends their median lifespan ~50%, in part by sensitizing *p53 *mutant cells to apoptosis in response to genotoxic stress [96]. These studies suggest that disruption of the circadian clock in itself does not compromise mammalian DNA repair and DNA damage checkpoints and does not predispose animals to spontaneous and ionizing radiation-induced cancers. The effect of circadian clock disruption on cellular response to DNA damage and cancer predisposition may depend on the mechanism by which the clock is disrupted, and elucidation of this mechanism warrants further investigation.

Another aspect of DNA damage response is DNA repair. Cells have evolved a number of mechanisms to repair damaged DNA. One such repair mechanism, nucleotide excision repair, is a multicomponent system that replaces a short single stranded region encompassing a DNA lesion. Recently, the effect of the circadian clock on nucleotide excision repair has been investigated in mice. Nucleotide excision repair is found to display prominent circadian oscillations in mouse brain reaching at its maximum in the afternoon/early evening hours and minimum in the night/early morning hours. The circadian oscillation of the repair capacity is caused at least in part by the circadian oscillations in the expression of DNA damage recognition protein xeroderma pigmentosum A (XPA) [97].

## Iterative alterations of lifestyle: clock -cancer connection

The clock-cancer connection has been investigated in studies of pilots, flight attendants, and shift workers who are more likely to have disrupted circadian cycles due to abnormal work hours. Incidence of breast cancer increases significantly in women working nightshifts, being higher among individuals who spend more years and hours per week working at night [98]. Exposure to light-at-night, including disturbance of the circadian rhythm, possibly mediated via the melatonin synthesis and clock genes, has been suggested as a contributing cause of breast cancer. Since working nightshifts is prevalent and increasing in modern societies, this exposure may be of public health concern, and contribute to the ongoing elevation in breast cancer risk [99-101]. Keith *et al*. propose that circadian rhythms could be more important than family history in determining breast cancer risk [102]. A pilot study in India showed that the risk of developing breast cancer in menopausal visually challenged women is very much lower as compared to sighted women in the similar age group suggesting a relationship between visible light and breast cancer risk [103]. Another study revealed that women working more than 20 years of rotating night shifts have a significantly increased risk of endometrial cancer. In stratified analyses, obese women working rotating night shifts had doubled their baseline risk of endometrial cancer compared with obese women who did no night work, whereas no significant increase was seen among non-obese women [104]. Observations from a cohort study of Air Canada pilots showed a significantly increased incidence rate of prostate cancer when compared with the respective Canadian population rates [105]. A similar cohort of Nordic pilots demonstrated that the relative risk of prostate cancer increases as the number of flight hours in long distance aircraft increases [106]. A significant association between rotating-shift work and prostate cancer incidence among Japanese male workers has also been found [107]. Incidence rate of acute myeloid leukemia (AML) has been reported to be significantly increased in a cohort of Air Canada pilots in comparison to respective Canadian population rates [108]. It has also been found that working a rotating nightshift at least three nights per month for 15 or more years may increase the risk of colorectal cancer in women [109]. Colon cancer patients who have maintained a regular pattern of rest and activity rhythms have shown a fivefold higher survival time than those who have chaotic circadian rhythms [110].

## Aberrant expression of clock genes in cancer

Several reports have revealed that clock genes are found to be deregulated in various cancers. In comparison with nearby non-cancerous cells, more than 95% of breast cancer cells reveal disturbances in the expression of the three *Per *genes attributable to methylation of the per gene promoters [111]. Moreover, a structural variation of the *Per3 *gene has been identified as a potential biomarker for breast cancer in pre-menopausal women [112]. Significantly decreased expression of *Per1 *has been observed between sporadic breast tumors and normal samples, as well as a further significant decrease between familial and sporadic breast tumors for both *Per1 *and *Per2 *suggesting a role for both in normal breast function [113]. It has been demonstrated recently that *Per2 *is endogenously expressed in human breast epithelial cell lines but is not expressed or is expressed at significantly reduced level in human breast cancer cell lines. Expression of *Per2 *in these breast cancer cells results in inhibition of cell growth and induction of apoptosis demonstrating the tumor suppressive nature of PER2. Moreover, PER2 activity is found to be significantly enhanced in the presence of its normal clock partner CRY2. Furthermore, *Per2 *expression in cancer cell lines is associated with a significant decrease in the expression of *Cyclin D1 *and an up-regulation of *p53 *[114]. The proliferation in ovarian cancer cells has been found to follow a cyclical pattern of peaks and troughs that is out of phase with the circadian rhythm in proliferation of normal tissues [115,116]. Recently, it has been reported that expression levels of *Per1*, *Per2*, *Cry2*, C*lock*, and *CKIε *in ovarian cancers are significantly lower than those in normal ovaries. On the contrary, C*ry1 *expression is highest followed by *Per3 *and *Bmal1 *[117]. Similarly, significantly decreased expression levels of *Per1 *as compared to paired non-tumour tissues, have been reported in endometrial carcinoma (EC). The decreased *Per1 *expression in EC is partly due to inactivation of the *Per1 *gene by DNA methylation of the promoter and partly due to other factors. This downregulation of the *Per1 *gene disrupts the circadian rhythm, which might favour the survival of endometrial cancer cells [118]. In another study, the promoter methylation in the *Per1*, *Per2*, or *Cry1 *circadian genes has been detected in about one-third of EC and one-fifth of noncancerous endometrial tissues of 35 paired specimens indicating possible disruption of the circadian clock in the development of EC [119]. Serum-shocked synchronized prostate cancer cells have been found to display disrupted circadian rhythms compared with the normal prostate tissue. *Per1 *is down-regulated in human prostate cancer samples compared to normal prostates. Moreover, over-expression of *Per1 *in prostate cancer cells has resulted in significant growth inhibition and apoptosis [120].

CCAAT/enhancer-binding proteins (C/EBPs) are a family of transcription factors that regulate cell growth and differentiation in numerous cell types. The results from a recent study suggest that *Per2 *is a downstream C/EBPα-target gene involved in acute myeloid leukemia (AML). Its disruption might be involved in initiation and/or progression of AML, as significantly reduced expression of *Per2 *has been noted in lymphoma cell lines as well as in AML patient samples [121]. The expression of *Per1*, *Per2*, *Per3*, *Cry1*, *Cry2*, and *Bmal1 *is significantly impaired in both chronic phase and blast crisis of chronic myeloid leukemia (CML) samples compared with those in normal samples. Although no mutations have been detected within the coding region of *Per3*, the CpG islands in its promoter are methylated in all the CML samples. Likewise, the CpG islands of *Per2 *are also methylated in 40% of cases [122]. Recently, *Cryptochrome1 *has been found to be a valuable predictor of disease progression in early-stage chronic lymphocytic leukemia (CLL) [123]. More recently, it has been reported that CRY1: PER2 expression ratio is independent prognostic marker in chronic lymphocytic leukemia [124]. There is also a case report showing that a patient with primary cerebral B-cell non-Hodgkin's lymphoma (NHL) has lost circadian control of sleep [125]. Moreover, genetic association and functional analyses suggest that the circadian gene *Cry2 *might play an important role in NHL development [126]. Several circadian related genes have been found to be under-expressed in pancreatic cancer indicating that pancreatic tumors have altered circadian rhythms [127]. Recently, *Per1 *has been identified as a candidate tumor suppressor, epigenetically silenced in nonsmall-cell lung cancer (NSCLC). *Per1 *expression has been found to be low in a large panel of NSCLC patient samples and in NSCLC cell lines compared to normal lung tissue. The down-regulation of *Per1 *expression is associated with hypermethylation of the *Per1 *promoter. Moreover, the study reveals that aberrant acetylation of *Per1 *promoter is also a potential mechanism for silencing *Per1 *in cancer [128]. More recently, *Bmal1 *has been reported to be transcriptionally silenced by promoter CpG island hypermethylation in hematologic malignancies, such as diffuse large B-cell lymphoma and acute lymphocytic and myeloid leukemias. It has been shown that BMAL1 epigenetic inactivation impairs the characteristic circadian clock expression pattern of certain genes including *c-Myc*, in association with a loss of BMAL1 occupancy in their respective promoters. Furthermore, the DNA hypermethylation-associated loss of BMAL1 also prevents the recruitment of its natural partner, the CLOCK protein, to their common targets, further enhancing the perturbed circadian rhythm of the malignant cells [129].

Epigenetic technologies in cancer studies are helping increase the number of cancer candidate genes and allow us to examine changes in 5-methylcytosine DNA and histone modifications at a genome-wide level. In fact, all the various cellular pathways contributing to the neoplastic phenotype are affected by epigenetic genes in cancer. They are being explored as biomarkers in clinical use for early detection of disease, tumor classification and response to treatment with classical chemotherapy agents, target compounds and epigenetic drugs [130]. The discovery of cancer-relevant gene silencing by epigenetic mechanisms is closely linked to epigenetic drug design and development. Application of epigenetic therapies in terms of developing drugs that block epigenetic events in cancer is one of the major courses of action that can influence the epigenetic yield. Demethylating agents namely 5-azacytidine (5-aza-CR) and 5-aza-2'-deoxycytidine (5-Aza-CdR) are the only cytidine analogues that have been approved by the U.S. Food and Drug Administration (FDA) for hematological malignancies in non-toxic doses [131,132].

## Cancer as a circadian rhythm related disorder

Different lines of evidence in mice and humans suggest that cancer may be a circadian-related disorder [133,134]. A number of studies by Filipski *et al*. indicate that the circadian clock of the host might play an important role in the endogenous control of tumor progression. SCN ablation or exposure to experimental chronic jetlag (CJL) caused alterations in circadian physiology and significantly accelerated tumor growth. CJL suppressed or altered the rhythms of clock gene and cell cycle gene expression in mouse liver. It increased *p53 *and decreased *c-Myc *expression, a result in line with the promotion of diethylnitrosamine-initiated hepatocarcinogenesis in jet-lagged mice. The accelerating effect of CJL on tumor growth is counterbalanced by the regular timing of food access over the 24 hours. Meal timing prevented the circadian disruption produced by CJL and slowed down tumor growth. In synchronized mice, meal timing reinforced host circadian coordination, phase-shifted the transcriptional rhythms of clock genes in the liver of tumor-bearing mice and slowed down cancer progression [135].

Recent findings suggest that circadian genes may function as tumor suppressors [133,136] at the systemic, cellular and molecular levels due to their involvement in cell proliferation [82,89], apoptosis [85,91], cell cycle control [69,70,82], and DNA damage response [70,86,97]. The genetic or functional disruption of the molecular circadian clock may result in genomic instability and accelerated cellular proliferation, two conditions that favor carcinogenesis [137]. Thus, aberrant expression of circadian clock genes could have important consequences on the transactivation of downstream targets that control the cell cycle and on the ability of cells to undergo apoptosis thus potentially promoting carcinogenesis.

It must be noted that contrary to epidemiological data, genetic data do not always show a positive correlation between the disruption of circadian clock and manifestation of cancer. A direct and simple answer to the question, why disturbances in circadian rhythms can cause cancer in some cases and not others, could be that desynchronization of phases attributable to abnormal working hours may produce a more profound and generalized effect on the pathophysiology of cancer than a single mutation. Thus, shift work and circadian mutation may have different impacts on physiological processes. Abnormal working hours leading to desynchronization of endogenous clock with the environment can affect overall clock-controlled physiological processes that can result in partial or complete phase shifts between physiology and behaviour depending upon the circumstances. On the other hand, a single mutation of any clock gene may disrupt the system at a particular state not always producing such a drastic effect as cancer because of the compensatory and redundant role of other genes. Another possible answer to the abovementioned question is based on the fact that a cell has a variety of options to illicit DNA damage response. The cell may go through growth arrest to permit DNA repair, and if damage is removed, the cell may restore its normal state. If the cell fails to repair the DNA damage, it can undergo apoptosis. Simultaneously, the cell can proliferate without elimination of mutations which will lead to neoplasia and tumorigenesis. In case of considerable damage, massive apoptosis may lead to disruption of tissue integrity, thus, the cell undergoes senescence while retaining its metabolic activity. The final outcome will depend on the type of the cell, various extracellular signals, and the functional status of the relevant intracellular pathways. The circadian genes may control some important steps of these pathways, thus, insufficiency of any particular clock gene will affect the specific pathway in which it is involved. This, in turn, will determine the final outcome of exposure to DNA damage. Evidence is being generated to show that deficiency of certain clock proteins favors the trigger to senescence. *Bmal1*^-/- ^and *Clock*^-/- ^mice show signs of premature aging, *Bmal1*^-/- ^mice naturally in life [138] and Clock^-/- ^mice after exposure to ionizing radiation [139]. Moreover, *Per2 *mutant mice show an increased number of senescent cells in vasculature developing early in life [140]. As stress-induced senescence has been proposed as one of the mechanisms for tumour suppression [141], the delay in tumorigenesis seen in *p53*^-/- ^*Cry1*^-/- ^*Cry*^-/- ^mice may indicate a switch of DNA damage response to senescence, which in these compound mutants is attributable to insufficiency of *Cryptochromes*.

## Cancer chronotherapy

Research in chronotherapy, which takes into consideration the biological time to improve treatments, plays an important role in devising new therapeutic approaches for the treatment of cancer [142]. The circadian timing system controls cellular proliferation as well as drug metabolism over 24 hours through molecular clocks, circadian physiology, and the SCN [143]. That is why both the toxicity and efficacy of more than 30 anticancer agents vary by more than 50% as a function of dosing time in experimental models [144]. The administration of a drug at a circadian time when it is best tolerated usually achieves best antitumor activity. This has been reported for antimetabolites, such as arabinofuranosylcytosine, 5-fluorouracil (5-FU), or 5-fluorouracil deoxyribonucleoside (FUDR); for intercalating agents such as doxorubicin; and for alkylating drugs such as melphalan or cisplatin [145]. The results obtained by numerical simulations of automaton model for the cell cycle indicate that the least cytotoxic patterns of 5-FU and l-OHP (oxaliplatin) circadian administration match those used clinically. The model also shows that continuous administration of 5-FU and l-OHP has the same effect as the most cytotoxic circadian pattern of drug delivery. Furthermore, the model helps to identify factors that may contribute to explain why temporal patterns corresponding to minimum cytotoxicity for a population of healthy cells could at the same time prove more cytotoxic toward a population of tumour cells [146]. The clinical relevance of chronotherapy is currently being investigated along these lines for the outcome of patients suffering from metastatic breast and pancreatic cancers. Multicenter clinical trials comparing chronomodulated versus conventional therapy are being planned for the adjuvant treatment of colorectal cancer and for head and neck and biliary duct cancers [146]. More phase III trials will be needed to firmly establish chronotherapy in medical oncology.

A recent study finds that wild-type and circadian mutant mice demonstrate striking differences in their response to the anticancer drug cyclophosphamide (CY). While the sensitivity of wild-type mice varies greatly, depending on the time of drug administration, *Clock *mutant and *Bmal1 *knockout mice are highly sensitive to treatment at all times tested. On the contrary, mice with loss-of-function mutations in *Cryptochrome *(*Cry1*^-/-^*Cry2*^-/- ^double knockouts) were more resistant to CY compared with their wild-type littermates. This indicates that sensitivity of chemotherapeutic drug cyclophosphamide (CY) is directly correlated with the functional status of the major circadian transactivation complex, suggesting that molecular determinants of sensitivity to CY may be directly regulated by CLOCK-BMAL1, which is based on a CLOCK-BMAL1-dependent modulation of target B cell responses to drug-induced toxicity [147]. As discussed earlier, nucleotide excision repair is found to display prominent circadian oscillations in mouse brain reaching at its peak in the afternoon/early evening attributable to circadian oscillation of XPA [97]. It is interesting to note that the peak of DNA repair activity coincide with the previously determined peak of animal's resistance to CY forming a background for important clinical applications. However, further research is required to know whether the damage caused by CY is repaired by nucleotide excision repair mechanism in vivo. Anticancer agents generally produce their cytotoxic effect in both malignant and normal tissues. If we know the circadian rhythm of DNA repair capacity of cancer and normal tissues, we can extrapolate that the most favorable time for drug administration will be when the excision repair activity is low in cancer tissues and when the repair activity is high in normal tissues. Multiple preclinical models with different clock properties are needed for the personalization of cancer chronotherapeutics and the prophecy of optimal chronomodulated drug delivery. The stages where chronotherapeutics will be incorporated into the development of new anticancer drugs will have to be defined, ranging from screening to clinical phases.

## Conclusion

Circadian regulation is important to maintain normal cellular functions, and a disruption of core clock genes can be damaging to the organism's overall well-being. The work is in progress to explicate the cascading interactions of networks of CCGs that connect the clockwork to the expressed rhythms. Results from several epidemiological and genetic studies have shown that disruption of circadian rhythm may lead to cancer. Contrary to this, some genetic data have also shown negative results for tumorigenesis in clock mutants when challenged with genotoxic stress. This indicates that the effect of circadian clock disruption on cellular response to DNA damage and cancer predisposition may depend on the mechanism by which the clock is disrupted and not on circadian dysregulation itself. However, overall the clock-cancer connection has gained some limited but consistent support from previous studies. Further Research is needed to reveal the mechanism behind the loss of circadian control which contributes to disease states at the organ and systemic levels. Finally, the circadian system may serve as a unique system for studying the mechanisms of cancer and for developing novel chronotherapeutic strategies to facilitate the treatment of cancer.

## Competing interests

The authors declare that they have no competing interests.

## Authors' contributions

SR and SM contributed equally for this review article (literature search, systematization and writing).
